# New Insights into the Adsorption Mechanism of Vanadium Through Quaternary Ammonium Salt-Functionalized SiO_2_: Synergistic Experiments Utilizing Energy Decomposition Analysis

**DOI:** 10.3390/molecules30071593

**Published:** 2025-04-02

**Authors:** Qiang Fu, Jianhua Tian, Jinjun Yang, Jie Wang, Meitong Li, Gangzhen Jiao, Yuhong Xie, Wenjiao Yuan, Cuihong Wang

**Affiliations:** 1 School of Environmental Science and Safety Engineering, Tianjin University of Technology, No. 391, Binshui Xi Road, Xiqing District, Tianjin 300384, China; f17695663750@163.com (Q.F.); tjyjj_2014@tjut.edu.cn (J.Y.); wj1247716961@163.com (J.W.); limeitong@email.tjut.edu.cn (M.L.); jiaogangzhen@tjut.edu.cn (G.J.); xieyuhong76@126.com (Y.X.); 2 Guangxi CNGR New Energy Science & Technology Co., Ltd., Qinzhou Port Area of China (Guangxi) Pilot Free Trade Zone, Qinzhou 535035, China; 15108085176@163.com; 3 School of Science, Tianjin Chengjian University, No. 26, Jinjing Road, Xiqing District, Tianjin 300384, China

**Keywords:** vanadium, adsorption mechanism, energy decomposition analysis, chemical bonds, non-bonding interactions

## Abstract

Introducing organic functional groups to adsorbent surfaces enhances vanadium adsorption, an effective strategy for vanadium enrichment. In a quest for a profounder comprehension of the above adsorption mechanism, this study synthesized five types of quaternary ammonium salt-functionalized silica (QAS-SiO_2_) and investigated the influence of functional groups, pH values, contact time, and temperature on vanadium (V) adsorption. The results indicated that the optimal QAS-SiO_2_ (SiO_2_@DMOA) achieved a vanadium adsorption rate of 99.40% and a maximum adsorption capacity of 39.16 mg g^−1^. SiO_2_@DMOA exhibited favorable adsorption selectivity for V over chromium (Cr), with a maximum separation factor (β_V/Cr_) of 135.42 at pH 3.3. SiO_2_@DMOA maintained efficient adsorption performance over five repeated cycles. A fusion of adsorption trials with energy decomposition analysis (EDA) tentatively unveiled that both chemical bonds and non-bonding interactions contributed to the interaction energy between organic functional groups and vanadium. Among them, chemical bonds accounted for 80.26%, while non-bonding interactions accounted for 19.74%. Based on EDA analysis, the interaction characteristics of different structural quaternary ammonium salts with vanadium in adsorption and extraction processes are discussed. Additionally, steric hindrance, the charge of the vanadium species, polarizability, and solvation effects, all played significant roles in the adsorption process.

## 1. Introduction

Vanadium (V), an important metallic element, finds indispensable utility in aerospace alloys, steel production, chemical catalysts, and energy materials [1,2]. It was reported that global V consumption was expected to soar to 130.1 kt by 2024 and increase with an annual growth rate of 5% [3]. However, V frequently coexists with over 70 other mineral metals (including iron (Fe), titanium (Ti), uranium (U), and aluminum (Al)) with a content of about 1%, giving rise to a difficulty in its separation and purification [1,2]. Moreover, the production process of the V-containing final products generates a substantial amount of V-based waste residues and effluents [4], resulting in the waste of the resource and environmental pollution. Herein, designing and developing materials and technologies for the efficient adsorption, separation, and regeneration of vanadium from residues and effluents holds significant scientific value and practical importance for environmental protection and sustainability.

To realize the enrichment and recovery of V, various techniques and methods have been reported, including chemical precipitation, ion exchange, solvent extraction, bioremediation, photocatalytic reduction, and membrane separation [5,6]. However, the abovementioned methods have some drawbacks, for example, high toxicity, high energy consumption, complicated processes, and high cost [7]. Owing to its high purification efficiency, low energy consumption, and eco-friendliness, adsorption technology has gained widespread application in diverse metal separation and recovery [8]. Recently, a great diversity of biomass materials (collagen fiber [9], chitosan [10], sawdust [11]), mineral materials (montmorillonite [12], kaolinite [13], and zeolite [14]), and industrial wastes [15,16] have been developed for V adsorption and recovery, but the strict activation condition and poor reusability are still bottlenecks limiting the industrial application. Thanks to its outstanding physiochemical nature (such as high specific surface area, hydrothermal stability, and a diversity of surface functionality) and cost effectiveness, SiO_2_ is attracting more and more interest [6]. Numerous SiO_2_-based composites modified with trypsin [17], tetrakis(4-carboxyphenyl)porphyrin [18], 2-methyl-8-quinolinol [8], thiol [19], and aminopropyl triethoxysilane [20] were synthesized and employed for vanadium adsorption, recovery, or detection. Due to the designability, adjustability, reactivity, versatility, and flexible nature of the organic functional groups, the incorporation of organic functional moieties has emerged as a potent strategy and calculated maneuver to enhance the efficacy and selectivity of metal adsorption.

**Scheme 1:**
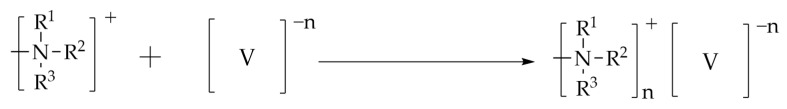
Formation of ion pairs between the quaternary ammonium salts and negatively charged vanadium species.

Considering their high efficiency in V extraction/separation/adsorption [21,22] and the diversity of their chemical structure, quaternary ammonium salts, as a crucial family of organic functional groups, have been used to modify the adsorbents and resins for the adsorption of V’s other pollutants [20,23,24,25]. The adsorption mechanisms are predominantly attributed to the electrostatic interaction, ion exchange, adsorption–reduction process, and formation of inner-sphere complexes [26]. Via electrostatic attraction and ion exchange, it is well understood that the nitrogen (N) in these salts bears a positive charge, facilitating the formation of ion pairs with negatively charged V species (Scheme 1). Nevertheless, the roles of the substituents R^1^, R^2^, and R^3^ attached to the N in the V adsorption process have seldom been discussed in depth, and, in particular, have not been quantitatively analyzed. In addition, it is noteworthy that the quaternary ammonium salts (such as Aliquat 336) can serve as highly efficient extractants to extract V from the V-bearing leachates [27,28,29,30]. Quaternary ammonium salt extractants usually have multiple long-chain alkyl substituents, with a key role of enhancing the extractant’s hydrophobicity, thereby facilitating the transfer of metal ions from the aqueous phase to the organic phase. To the best of our knowledge, there is little discussion about the roles of the functional groups decorated on the N atom (for example, the long-chain or short-chain alkyl substituent enhances metal binding). Adsorption and extraction of vanadium both entail interactions between surface-bound organic functional groups or organic extractants and vanadium species. However, detailed discussions of the structural distinctions between these organic components are uncommon.

In this work, we synthesized five types of silica gels modified by quaternary ammonium salts with different chemical structures (organic functional groups) and investigated their adsorption effect toward V(V) under various conditions (such as pH, contact time, ion strength, and presence or absence of the Cr(VI)). In addition, the reusability of quaternary ammonium salt-functionalized silica gels was also investigated via adsorption–desorption experiments. Among these five organic functional groups, three are linear alkyl chains with various chain lengths, and the other two are ethanolamine-based fragments. The V(V) extraction and adsorption is closely correlated with the intrinsic structural nature. The existing explanations pertaining to vanadium adsorption predominantly encapsulate and generalize the prominent features of adsorption, yet they lack insights into the inherent nature of interactions between organic functional groups and vanadium. To transcend these limitations posed by the “phenomenology”, we present a quantitative analysis of the intricate interactions between organic functional groups and the V ion (including both chemical bonds and non-bonding) via energy decomposition analysis (EDA), aiming to provide a new approach or perspective to understand and predict the molecular interaction, which is difficult to directly determine in experiments. This work is helpful to deepen our understanding of the interaction mechanism between quaternary ammonium salts and transition metal ions, and is expected to guide the design and synthesis of related materials in the future.

## 2. Results and Discussion

### 2.1. Characterization

#### 2.1.1. Fourier Transform Infrared (FTIR) Spectroscopy

**Figure 1:**
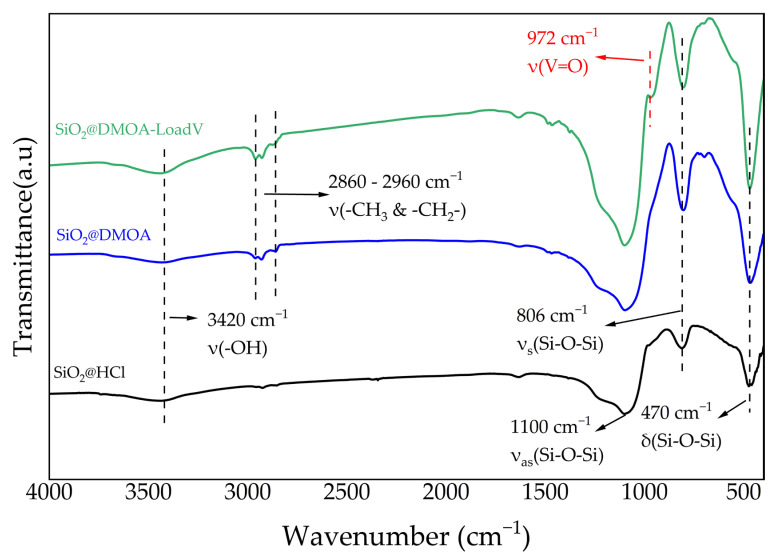
FTIR of SiO_2_@HCl, SiO_2_@DMOA and V-loaded SiO_2_@DMOA.

FTIR spectra of SiO_2_@HCl, SiO_2_@DMOA, and V-loaded SiO_2_@DMOA (SiO_2_@DMOA-V) are shown in Figure 1. The peak at 3420 cm^−1^ corresponds to -OH stretching vibrations due to physically adsorbed water and surface hydroxyl groups of the silica [31]. The region between 2860 cm^−1^ and 2960 cm^−1^ is attributed to methyl (-CH_3_) and methylene (-CH_2_-) stretching vibrations [32]. The peaks at 1100 cm^−1^ and 806 cm^−1^ correspond to asymmetric and symmetric stretching vibrations of Si-O-Si bonds, respectively, while the peak at 470 cm^−1^ is due to Si-O-Si bending vibrations [31,32]. A weak peak at 972 cm^−1^ is assigned to the V=O functional group of the SiO_2_@DMOA-V [33]. This confirms the successful functionalization of SiO_2_@DMOA and its adsorption of the V(V).

#### 2.1.2. Scanning Electron Microscopy (SEM)

**Figure 2:**
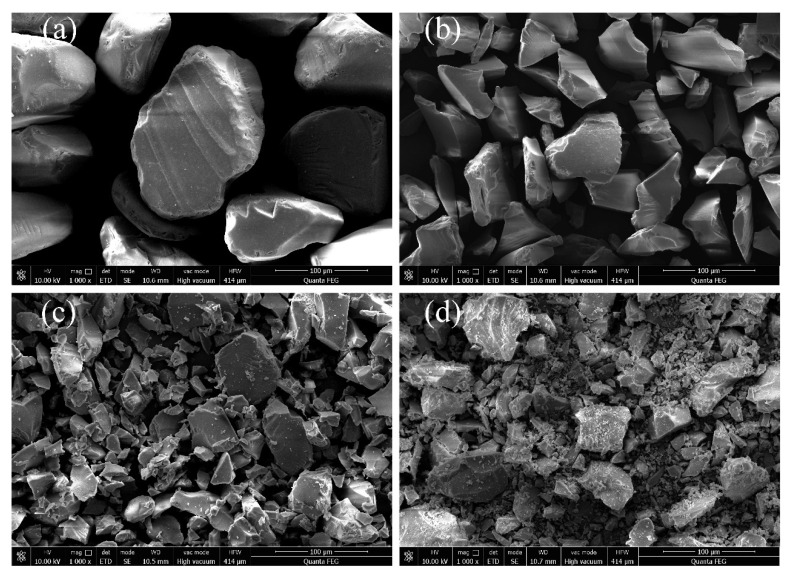
SEM images of (**a**) SiO_2_@HCl, (**b**) SiO_2_@CPTES, (**c**) SiO_2_@DMOA, and (**d**) SiO_2_@DMOA-V.

SEM images of SiO_2_@HCl, SiO_2_@CPTES, SiO_2_@DMOA, and SiO_2_@DMOA-V are shown in Figure 2. It can be clearly seen that the size of the bulks reduced and the roughness of the surface of the bulk increased upon modification by the functional groups. In particular, after the adsorption of the V(V) into the SiO_2_@DMOA, a large quantity of small particles aggregated on the surface of the bulk. The substantial differences in physical structure before and after V(V) loading on SiO_2_@DMOA revealed that the pores on the surface were filled with smaller particles (of aggregated V(V)), resulting in a rougher surface. This could be attributed to the V(V) being anchored to the surface by functional groups. The atom percentages of the Si, O, C, N, Cl and V (measured by EDS) are listed in Table S1. The results indicate successful preparation of SiO_2_@CPTES, which contains 35.12% carbon and 2.16% chlorine, in comparison to SiO_2_@HCl. SiO_2_@DMOA shows 4.96% nitrogen, higher than SiO_2_@CPTES, implying successful modification with *N*,*N*-dimethyloctylamine on the silicon gel surface and confirming SiO_2_@DMOA preparation. Additionally, SiO_2_@DMOA-V displays 1.45% vanadium versus SiO_2_@DMOA, confirming the adsorption of V on the surface.

#### 2.1.3. Time-of-Flight Mass Spectrometry (TOF-MS)

There are various forms of V in aqueous solutions, influenced by several factors such as concentration, pH, and ionic strength. Various monomeric and polymeric oxo-vanadate species, and tetramers (V_4_ species) and decamers (V_10_ species), were reported [34]. It is reported that complicated forms of V in aqueous solutions significantly affect adsorption efficiency [35].

TOF-MS was utilized to investigate the form of the V ion at different pHs (from pH = 1 to 6) [36]. The TOF-MS spectra of vanadium-containing solution (2 × 10^−3^ M) at pH = 3 and other pH values are presented in Figures S1–S6. In the pH range of 1~2, the predominant forms of V were the dimers (V_2_, *m*/*z*: 152.88–199.81) and tetramers (V_4_, *m*/*z*: 216.83–555.27). At pH = 3~6, the main species was V_10_ at *m*/*z*: 462.66 since V_2_ and V_4_ species can transform into V_10_ species (Equations (1)–(3)) [34]. Under optimal adsorption conditions, vanadium primarily existed in the form of V_10_ in the solution and was adsorbed by SiO_2_@DMOA.

(1) $$4H_{2}VO_{4}^{-}+3H^{+}↔HV_{4}O_{11}^{-}+5H_{2}O$$

(2) $$2HV_{2}O_{6}^{-}+H^{+}↔HV_{4}O_{11}^{-}+H_{2}O$$

(3) $$5HV_{4}O_{11}^{-}+H^{+}↔2V_{10}O_{26}^{2-}+3H_{2}O$$

### 2.2. Screening of Optimal QAS-SiO_2_

**Figure 3:**
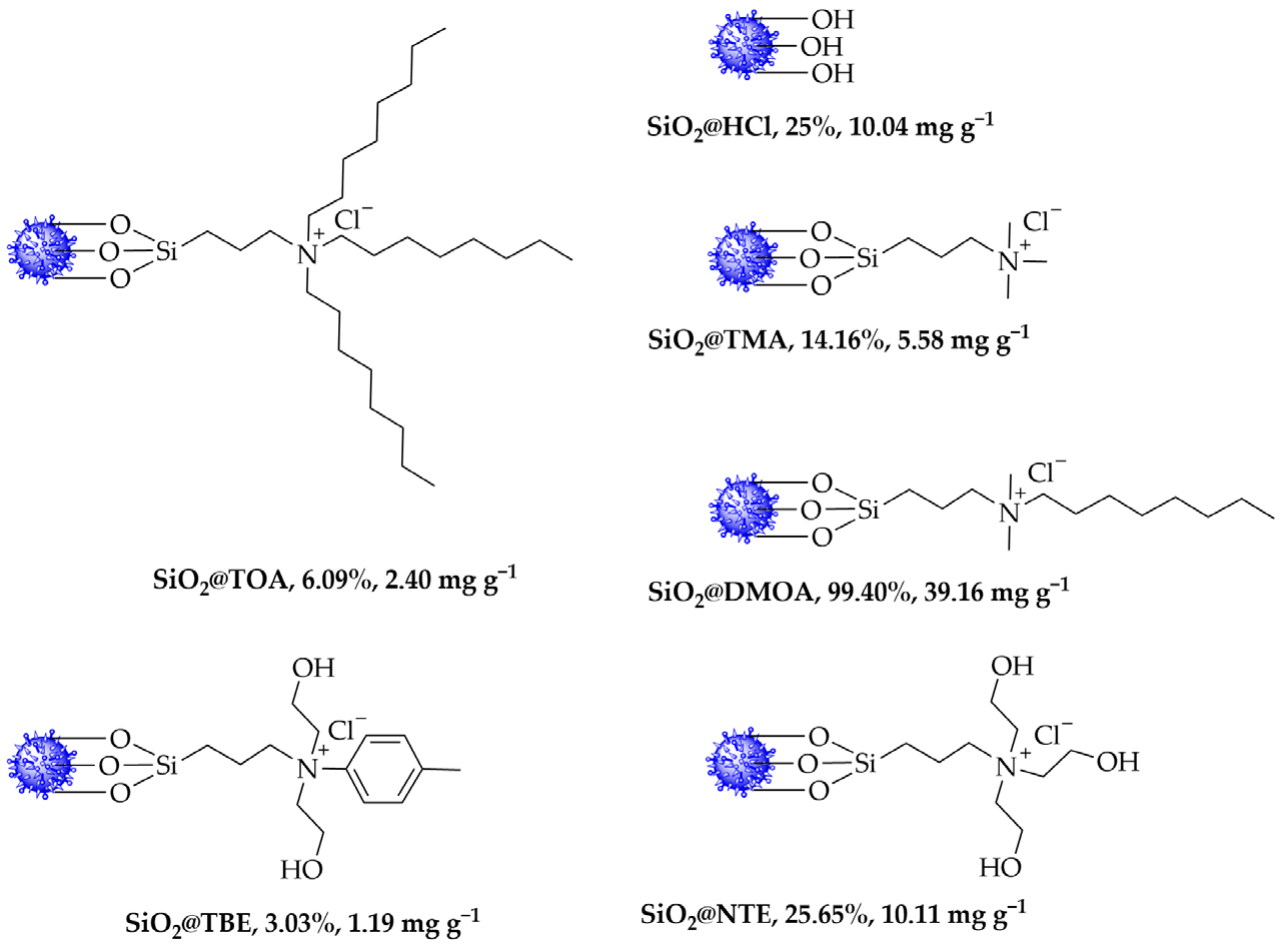
The V Adsorption rate (%) and q_e_ (mg g^−1^) of five QAS-SiO_2_ adsorbents.

Variations in the affinity of adsorbent structures can influence the efficiency of the adsorption of the metal. In this work, five QAS-SiO_2_ absorbents (as shown in Figure 3) were prepared with different carbon chains, aromatic hydrocarbon groups, and hydroxyl-containing groups. A previous report indicated that due to a low V content in leaching solutions, ammonium salts as extractants exhibited optimal removal efficiency only at pH = 3 [37]. Separately, 50 mg of SiO_2_@DMOA, SiO_2_@TOA, SiO_2_@TBE, SiO_2_@NTE, and SiO_2_@TMA were mixed with vanadium(V) solution (2 × 10^−3^ M, pH = 3) at room temperature for a period of 12 h to adsorb. Based on the adsorption performance, the material with the highest adsorption capacity was selected for further in-depth investigation. As demonstrated in Figure 1, the adsorption rate and q_e_ of SiO_2_@NTE and SiO_2_@TMA were 25.65% and 10.11 mg·g^−1^, and 14.16% and 5.58 mg·g^−1^, respectively. For SiO_2_@TOA and SiO_2_@TBE, lower values were exhibited of 6.09% and 2.40 mg g^−1^, and 3.03% and 1.19 mg g^−1^, respectively. It is noteworthy that SiO_2_@DMOA demonstrated a superior adsorption rate (99.40%) and adsorption capacity (39.16 mg g^−1^), which were significantly higher than those of the other four absorbents. The remarkable difference is probably attributed to the structural variation of the absorbent and the existing form of the V(V) at pH 3, which will be discussed in depth later. For brevity, only the experimental data of SiO_2_@DMOA are presented.

### 2.3. Effect of the pH on Adsorption Efficiency

**Figure 4:**
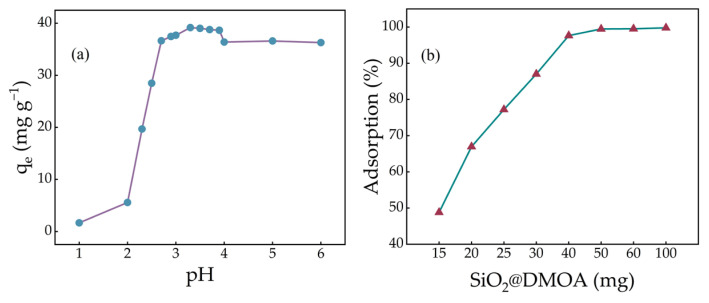
(**a**) Effect of pH on the adsorption of the V(V) in SiO_2_@DMOA and (**b**) the adsorption effect as a function of the dosage of SiO_2_@DMOA.

Solution pH is a crucial factor to affect adsorption efficiency [38]. The results in Figure 4a demonstrate that as the pH increased from 2 to 3, q_e_ enhanced from 5.58 mg·g^−1^ to 37.70 mg·g^−1^, followed by a decrease to 36.39 mg g^−1^ at pH 4. This indicates an effective V adsorption range of pH 2–4. Further investigation was carried out within the pH range of 2.3–3.9, which revealed a continuous increase in q_e_ from 19.68 mg·g^−1^ at pH 2.3 to the maximum value of 39.16 mg·g^−1^ at pH 3.3. This may be associated with the different chemical forms of the V(V) in aqueous solutions.

### 2.4. Optimal Dosage of SiO_2_@DMOA

A given amount of SiO_2_@DMOA (15–100 mg) was mixed with the V(V) solution (20 mL, 2 × 10^−3^ M, pH 3.3) and allowed to adsorb for 12 h. Figure 4b suggests that the adsorption rate of the V(V) increased from 48.81% (15 mg dosage) of SiO_2_@DMOA to 99.40% (50 mg dosage). With increasing the dosage of the SiO_2_@DMOA, the V(V) adsorption reached an equilibrium value or a saturate state, because no more adsorption sites were available. Therefore, 50 mg of SiO_2_@DMOA was selected as the optimal dosage. The dosage of the adsorbent was only 0.25% of the vanadium-containing water.

### 2.5. Effect of Contact Time

**Figure 5:**
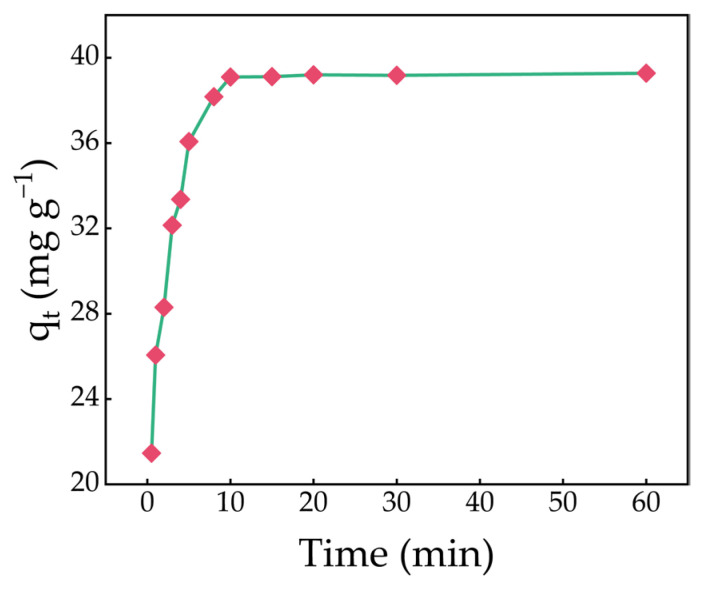
Effect of contact time on adsorption efficiency of V(V) in SiO_2_@DMOA.

In order to evaluate the impact of contact time on the adsorption process, a mixture of 50 mg SiO_2_@DMOA and the V(V) solution (20 mL, 2 × 10^−3^ M, pH = 3.3) was used for the adsorption experiment and the contact time was set from 1 to 60 min. As shown in Figure 5, the adsorption rate rapidly increased to 7.21 mg·g^−1^·min^−1^ within the first 5 min, which suggests abundant active adsorption sites on SiO_2_@DMOA’s surface [39]. Subsequently, the rate slowed to 0.32 mg·g^−1^·min^−1^ between 5 and 15 min. After the adsorption of 15 min, adsorption reached an equilibrium state with q_e_~39.16 mg·g^−1^, in line with a previous report on metal adsorption kinetics [38].

### 2.6. Adsorption Kinetics

**Figure 6:**
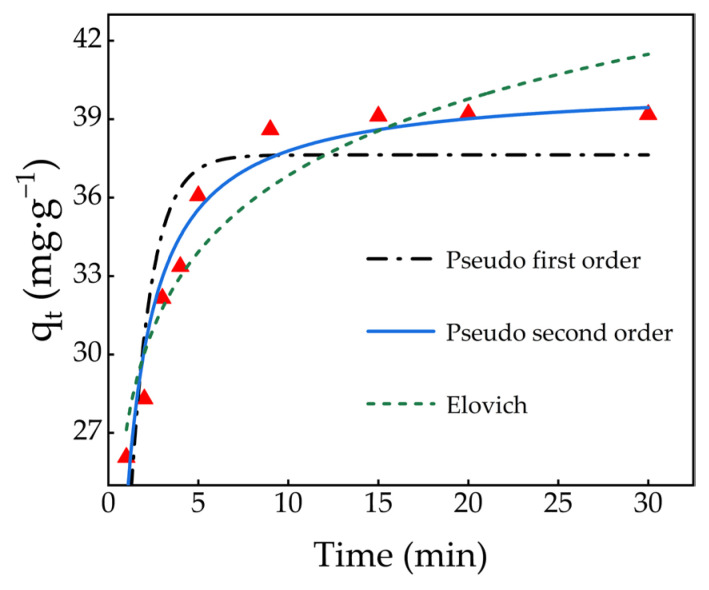
Pseudo-first-order, pseudo-second-order, and Elovich models used for V(V) fitting of adsorption process.

To elucidate the adsorption kinetics of V(V) in the presence of SiO_2_@DMOA, nonlinear regression analysis was performed using pseudo-first-order (PFO), pseudo-second-order (PSO), and Elovich models [40], as presented in Equations (4)–(6).

(4) $$q_{t}=q_{e}\left(1-{\mathrm{e}}^{K_{1}t}\right)$$

(5) $$q_{t}=\frac{K_{2}q_{e}^{2}t}{1+Kq_{e}t}$$

(6) $$q_{t}=\frac{1}{\beta }\ln\left(1+\alpha \beta t\right)$$

where *q*_e_ (mg·g^−1^) and *q*_t_ (mg·g^−1^) represent the adsorption capacity at equilibrium and the given time (t, min), respectively. *K*_1_ (min^−1^) and *K*_2_ (g·mg^−1^·min^−1^) are the pseudo-first order and pseudo-second order model constants, respectively. *α* (mg·g^−1^·min^−1^) is the initial adsorption rate constant, and *β* (g mg^−1^) is a constant related to the extent of surface coverage and activation energy of chemical adsorption. The fitting curves and detailed parameters are shown in Figure 6 and Table 1, respectively. In Table 1, the coefficient of determination (R^2^) and the root mean square of the error (RMSE) were used to assess the goodness of fit with the experimental data [41].

Among the models, PSO (R^2^ = 0.937, RMSE = 1.259) demonstrated a better fit than PFO (R^2^ = 0.709, RMSE = 2.701), indicating chemical adsorption of V(V) occurred on SiO_2_@DMOA. The parameters of the Elovich model, *α* (2599.132) and *β* (0.237) also suggest a chemical reaction between SiO_2_@DMOA and V(V) in a short time frame [42].

To explore the diffusion mechanism, intraparticle diffusion and Boyd’s models were employed to describe in detail the adsorption process, as shown in Equations (7)–(9) [41]:

(7) $$q_{t}=K_{\dot{i}d}t^{\frac{1}{2}}+C$$

(8) $$B_{t}=-0.4977-\ln\left(1-F\right),For\;F(t)>0.85$$

(9) $$B_{t}=\left(\sqrt{\pi }-\sqrt{\pi -\frac{\pi^{2}F}{3}}\right),For\;F\left(t\right)\leq 0.85$$

where *K*_id_ (mg·g^−1^·min^1/2^) is the intraparticle diffusion rate constant, and *C* is a constant estimating the thickness of the boundary layer. *B*_t_ is Boyd’s number, and *F*(t) can be expressed as *q_t_/q*_e_ at a specific time *t*.

**Figure 7:**
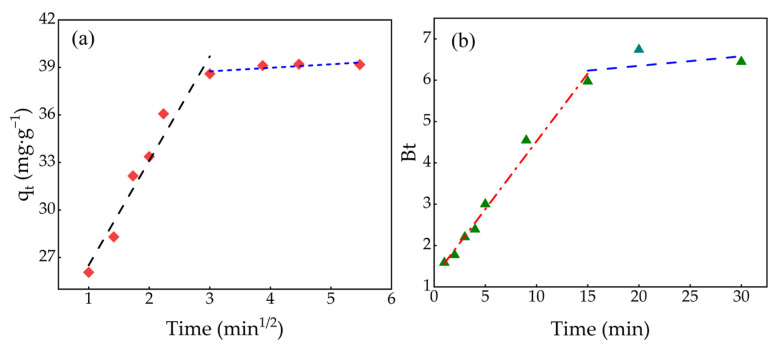
(**a**) Intraparticle diffusion model and (**b**) Boyd’s model for the adsorption of V(V) onto SiO_2_@DMOA.

The particle intraparticle diffusion model was fitted to the experimental data, yielding the corresponding parameters (Figure 7a and Table 1). Comparison of slopes (*K*_i,1_ = 0.943 > *K*_i,2_ = 0.490) and intercepts (*C*_2_ = 38.08 > *C*_1_ = 19.91) revealed two linear plots, indicating multiple steps influencing the sorption process [41]. These include film diffusion of V(V) on the SiO_2_@DMOA surface and intraparticle diffusion within the particles [43], with film diffusion showing a higher rate. The change in intercept is due to the gradual decrease in adsorbate concentration on the adsorbent surface over time, leading to a reduction in the mass transfer rate to the adsorbent.

Through analysis using Boyd’s model (Figure 7b), the determining factor of the rate-controlling step in the adsorption process can be discerned. If the fitting result is not a straight line but a curve, it indicates that film diffusion may be a primary rate-limiting step [44].

In summary, based on the fitting results of the adsorption model, both film diffusion and intraparticle diffusion contribute to the adsorption process. However, film diffusion emerges as the dominant rate-limiting factor, which plays a decisive role in governing the entire adsorption process.

### 2.7. Adsorption Isotherm

The adsorption isotherm can be utilized to investigate the equilibrium state of V(V) adsorption onto SiO_2_@DMOA at a specific temperature and elucidates the distribution of V(V) between the liquid and solid phases. The Freundlich and Langmuir adsorption models were employed to establish the adsorption isotherms, respectively (Equations (10) and (11)) [40].

(10) $$q_{e}=\frac{q_{m}K_{L}C_{e}}{1+K_{L}C_{e}}$$

(11) $$q_{e}=K_{F}C_{e}^{\frac{1}{n}}$$

where *q*_e_ (mg·g^−1^) and *q*_m_ (mg·g^−1^) represent the experimental adsorption capacity and the maximum adsorption capacity, respectively, and *C*_e_ (mg·L^−1^) is the equilibrium concentration of V(V) in the solution. *K*_F_ (L·g^−1^) and n are the Freundlich constants related to adsorption capacity and energy, and *K*_L_ (L·mg^−1^) is the Langmuir constant associated with adsorption energy.

**Figure 8:**
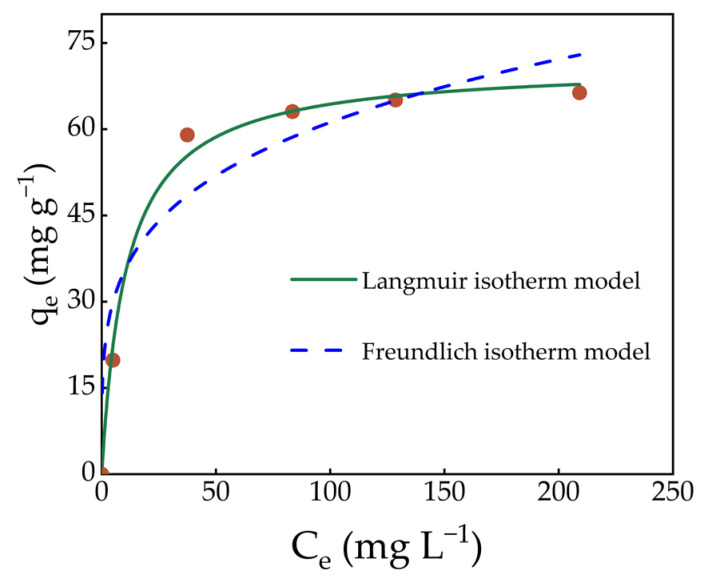
Freundlich and Langmuir isotherm models for V(V) adsorption onto SiO_2_@DMOA.

The results of the nonlinear regression are depicted in Figure 8, and the fitting parameters are presented in Table 2. Comparing the Langmuir model to the Freundlich model, the Langmuir model exhibits a higher correlation coefficient (R^2^) and a lower root mean square error (RMSE), which suggests a better fit to the experimental data. This indicates that the adsorption of V(V) onto SiO_2_@DMOA occurred predominantly through a monolayer chemical adsorption process [45].

The efficiency of the predicted adsorption process can be expressed using the dimensionless separation factor *R*_L_ associated with the Langmuir model (Equation (12)) [41].

(12) $$R_{L}=\frac{1}{1+K_{L}C_{o}}$$

where *C_o_* (mg·g^−1^) is the initial concentration of V(V), and *K*_L_ (L mg^−1^) is the Langmuir constant related to adsorption energy.

By conducting nonlinear fitting with *C_o_* as the variable, the results can be interpreted based on the shape of the fitted curve and the *R*_L_ value. Figure S7 shows that the curve is concave and *R*_L_ is 0.40~0.028, which indicates a favorable adsorption process [46]. *R*_L_ dropped with an increase in *C_o_*, revealing that SiO_2_@DMOA is more inclined to absorb high concentrations of V(V) [41].

### 2.8. Adsorption Thermodynamics

**Figure 9:**
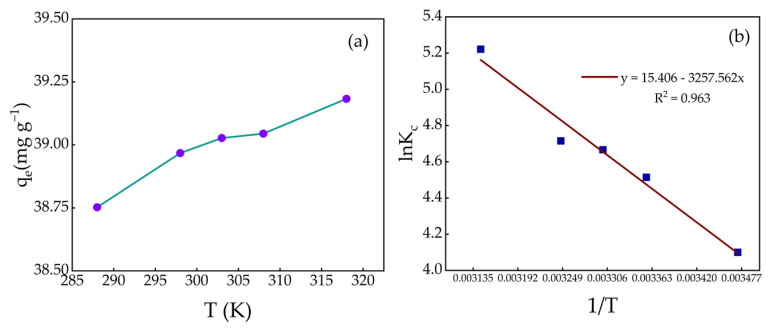
(**a**) q_e_ as a function of temperature in the adsorption process of V (V) onto SiO_2_@DMOA and (**b**) Van’t Hoff plot in the temperature range of 288–318 K.

A mixture of SiO_2_@DMOA (50 mg) and V(V) solution (20 mL, 2 × 10^−3^ M, pH = 3.3) was used to investigate the effect of the temperature on the adsorption efficiency in a temperatures range of 288~318 K for 30 min. In Figure 9a, the results reveal that q_e_ increased with increasing the temperature, and reached 39.16 mg·g^−1^ at 318 K. Reduction in the boundary layer thickness around the adsorbent is probably ascribed to the decrease of mass transfer resistance and enhancement of diffusion of V(V) within the pores of the adsorbent [47].

As shown in Figure 9b, the Gibbs free energy (ΔG°), enthalpy (ΔH°), and entropy (ΔS°) can be calculated using the plot of the lnKc vs. 1/T (Equations (13)–(15)) [41].

(13) $$K_{c}=\frac{C_{o}-C_{e}}{C_{e}}$$

(14) $$\ln K_{c}=\frac{-\Delta H^{o}}{RT}+\frac{\Delta S^{o}}{R}$$

(15) $$\Delta G^{o}=\Delta H^{o}-T\Delta S^{o}$$

where *K_c_* is the thermodynamic equilibrium constant; *C_o_* and *C_e_* (mg L^−1^) represent the initial and equilibrium concentrations of V(V), respectively. *R* (8.314 J·mol^−1^·K^−1^) is the gas constant and *T* (K) is the temperature of the adsorption process.

The thermodynamic parameters for the adsorption of V(V) onto SiO_2_@DMOA are summarized in Table 3. ΔH° = 27.08 kJ·mol^−1^ (>0) indicates an endothermic adsorption process. ΔS° = 128.08 J mol^−1^ K^−1^ (>0) suggests an increase in randomness at the solid–liquid interface during the adsorption process, that is, the heavy metal ions near the surface of the adsorbent were more disordered than before adsorption, and the amount bound to the adsorbent increased [48]. The negative ΔG° value indicates that the adsorption process was spontaneous, and the decrease in ΔG° with increasing temperature suggests a more favorable adsorption process at higher temperatures, which is consistent with previous reports [49].

### 2.9. Effect of Ionic Strength

**Figure 10:**
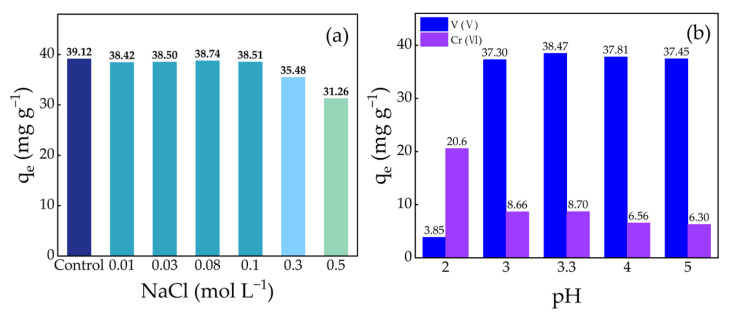
(**a**) Effect of NaCl on adsorption of V(V) onto SiO_2_@DMOA and (**b**) effect of solution pH on adsorption of V(V) and Cr(VI) by SiO_2_@DMOA.

In the V-containing wastewater, other ions (with a high concentration) might affect the structural form of the V(V) or the binding of the adsorbent with the V(V). To investigate this, 50 mg of SiO_2_@DMOA was added to V(V) solutions (20 mL, 2 × 10^−3^ M, pH = 3.3) with varying NaCl concentrations (0.01, 0.03, 0.08, 0.10, 0.30, 0.50 M) and allowed to adsorb for 30 min. The results in Figure 10a reveal that the adsorption capacity changed little with an increase in the concentration of the NaCl from 0 to 0.1 M·L^−1^, but followed by a gradual reduction with further increasing the concentration to 0.5 M·L^−1^. That is, the impact was less significant at low NaCl concentrations (<0.1 M). A similar result was reported, where the effect of NaNO_3_ was noticeable above 0.1 M in the V(V) adsorption process using the silica dioxide as an adsorbent [50]. Excessive ions could potentially alter the activity of the V(V) and slightly increase electrostatic repulsion, affecting the affinity of SiO_2_@DMOA functional groups with the V(V) ions [51,52].

### 2.10. Separation of the V and Cr

Due to the similarity in physicochemical properties of V and Cr, their separation still is a challenge [53]. Based on the result above, SiO_2_@DMOA (50 mg) was added into a mixed solution (20 mL, pH of 2–5) with V(V) (2 × 10^−3^ M) and Cr(VI) (1.63 × 10^−3^ M) for investigating the separation of V and Cr. As shown in Figure 10b, a maximum separation coefficient β_V/Cr_ (135.42) was observed at pH 3.3. Decreasing pH from 3 to 2 resulted in a decrease in q_e_ from 38.47 mg·g^−1^ to 3.85 mg·g^−1^, while q_e_ for Cr(VI) increased from 8.70 mg·g^−1^ to 20.60 mg·g^−1^. This may arise from different nucleophilicities of oxygen-containing metal anions. Polymeric vanadates often possess a greater negative charge and larger thermodynamic radius compared to chromates, leading to higher polarization and nucleophilicity of vanadates [35]. At pH = 2, q_e_ of the V(V) (3.85 mg·g^−1^) was lower than that of the Cr(VI) (20.60 mg·g^−1^), indicating that pH influences the chemical species of the V(V) in the solution. It was reported that there is predominantly VO_2_^+^ at pH < 2, and it would transform into polynuclear anions at pH = 2.5~6 [5]. For Cr(VI), the primary species is HCrO_4_^−^ with a concentration lower than 2 mM and at pH = 1~6 [54]. The different forms of metal anions in aqueous solution are the key to understanding the discrepancy in selective adsorption of the V(V) and Cr(VI) [55].

### 2.11. Desorption and Regeneration

**Figure 11:**
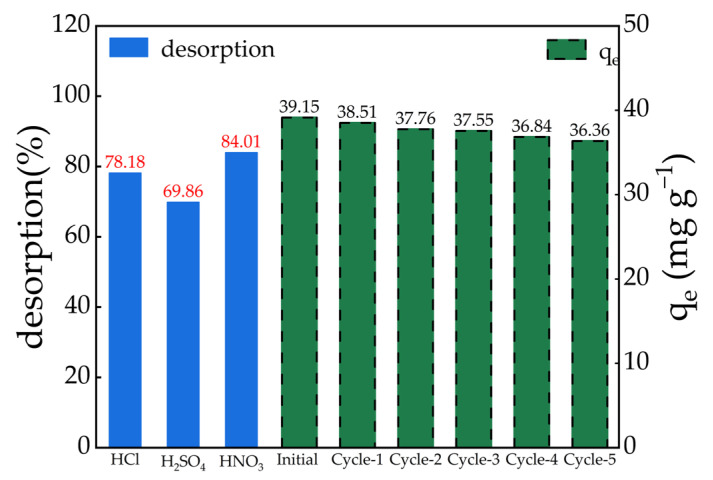
Effect of different desorbents on the desorption of SiO_2_@DMOA and the V(V) adsorption after regeneration of SiO_2_@DMOA using the HNO_3_ as desorption agent.

Adsorption–desorption experiments were conducted to evaluate the reusability of SiO_2_@DMOA. The adsorption–desorption results are depicted in Figure 11. To determine the optimal desorption agent, the V-loaded SiO_2_@DMOA was subjected to a single desorption using 1 M HCl, H_2_SO_4_, and HNO_3_, separately. The results indicated that HNO_3_ showed the highest desorption efficiency (84.01%) followed by HCl (78.18%) and H_2_SO_4_ (69.86%). Subsequent adsorption–desorption experiments were carried out using HNO_3_ as the desorption agent. After two cycles, q_e_ slightly decreased from 39.15 mg·g^−1^ (initial) to 36.36 mg·g^−1^ (cycle-5), probably due to incomplete release of active sites during the desorption or small portion of V(V) remaining in the pores of SiO_2_@DMOA. This could arise from the formation of ionic bonds between V(V) and SiO_2_@DMOA’s active sites [41]. Additionally, dissolution in the desorption agent and minor mass loss of SiO_2_@DMOA may contribute to the slight reduction in q_e_.

### 2.12. Adsorption Mechanisms

Intuitive bonding models based on the principles of quantum mechanics are widely adopted to explore the experiment results and phenomena. However, it is still a challenge to obtain a better and in-depth understanding of the intrinsic nature of the material and prediction of the dynamic process because these models depend significantly on the parameters (which are temporarily set) and are probably in the absence of a firm theoretical foundation. Therefore, a more precise and effective method or mechanism which can integrate the quantum mechanics should be developed to further assist in the sole empirical expectation or observation [56]. In this work, the ion exchange used to delineate the adsorption mechanism is not enough because it falls short in providing a comprehensive insight into chemical bonding and the non-bonding interactions. The energy decomposition analysis (EDA), which was developed by Morokuma [57] and Ziegler and Rauk [58], is a powerful method for bridging the gap between elementary quantum mechanics and the nature of the chemical bond and non-bonding interactions. Via EDA, the instantaneous interaction energy Δ*E*_int_ between two components A and B in a molecule (A–B) can be divided into three well-defined parameters, which are shown as follows: Δ*E*_elstat_, the quasi-classical electrostatic interaction energy between the charge densities of the components; Δ*E*_Pauli_, the exchange repulsion between the components based to Pauli’s principle; and Δ*E*_orb_, the energy gain due to orbital mixing of the components. These three parameters are defined by assigning intermediate states of the entire system during the course of bond formation, which are calculated by the laws of quantum mechanics. Current EDA-derived methods can be classified into three subgroups [59], the variation-based EDA method, perturbation-based EDA method, and real space-based EDA method, which have been employed to characterize, quantify, and interpret interactions between components of quantum systems [56,60]. In this work, a method based on dispersion-corrected density functional theory (DFT), known as sobEDA, is utilized to shed light on the essence of interactions during adsorption processes. The sobEDA methodology combines the quantum chemistry program Gaussian and the wavefunction analysis program Multiwfn to perform sobEDA energy decomposition, and has demonstrated its efficacy in weak interactions, chemical bonding interactions, open-shell configurations systems, and the interplay among diverse molecular components [61].

The total interaction energy Δ*E*_int_ in the sobEDA model can be divided into the following physical parameters, as shown in Equation (16):

(16) $$\Delta E_{int}=\Delta E_{els}+\Delta E_{x}+\Delta E_{DFT_{c}}+\Delta E_{dc}+\Delta E_{rep}+\Delta E_{orb}$$

Δ*E*_els_: Classical electrostatic interaction energy between components (with positive or negative value).

Δ*E*_x_: Exchange interaction energy (with a negative value), which reflects the contribution of exchange-related interaction energy between components.

Δ*E*_DFTc_: DFT correlation energy (DFTc stands for DFT correlation and the value is generally negative), which reflects the contribution of Coulomb-related interaction energy between components).

Δ*E*_rep_: Pauli repulsion energy (with a positive value), which reflects the energy increase caused by electron repulsion between different components to satisfy the Pauli exclusion principle.

Δ*E*_orb_: Orbital interaction energy (with a negative value), which comes from the energy change caused by the mixing of occupied and unoccupied orbitals within and between components, physically reflecting the overall impact of electron distribution polarization within components and electron transfer between components on energy. Covalent interactions are also included in this parameter.

Δ*E*_dc_: Dispersion correction energy (with a negative value).

**Figure 12:**
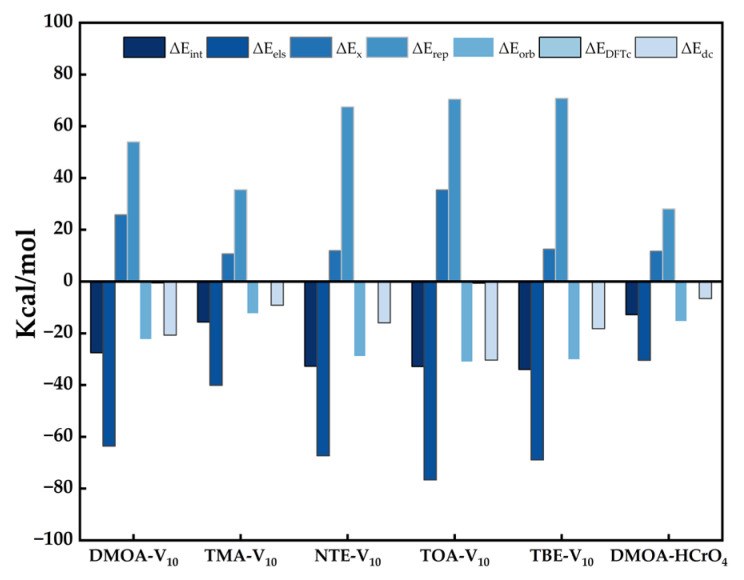
Interaction energy of five functionalized SiO_2_ forms loaded with V.

In Table 4 and Figure 12, Δ*E*_int_ values of five functionalized-SiO_2_ loaded with V are negative, affirming the existence of an adsorptive interplay. Δ*E*_int_ of five quaternary ammonium salts with V_10_ species (V_10_) ranks in the following order: TBE-V_10_ > TOA-V_10_ > NTE-V_10_ > DMOA-V_10_ > TMA-V_10_. Notably, no obvious difference could be seen among Δ*E*_int_ of TBE, TOA, and NTE. The longest molecular chain and highest molecular weight resulted in a high *E*_int_ of the SiO_2_@TOA. The high *E*_int_ of the SiO_2_@NTE and SiO_2_@TBE is probably mainly attributed to the multiple -OH groups. SiO_2_@TOA, SiO_2_@DMOA, and SiO_2_@TMA exhibit a similarity in their functional groups (alkyl group), as demonstrated in Figure 3. Generally, the alkyl group with a longer molecular chain shows a higher Δ*E*_int_. Therefore, the SiO_2_@TOA (with three octyl groups) presented the highest Δ*E*_int_, followed by SiO_2_@DMOA (with one octyl group) and SiO_2_@TMA (with three methyl groups). It is expected that a quaternary ammonium salt with a longer carbon chain or more -OH groups frequently exhibits a higher Δ*E*_int_ with V_10_. However, this experimental result is not in agreement with our expectation. As shown in Table 4 the qe values of these five quaternary ammonium salts in the adsorption process are ranked as follows: DMOA-V_10_ > NTE-V_10_ > TMA-V_10_ > TOA-V_10_ > TBE-V_10_. During the construction of functionalized silica dioxide models in sobEDA, the oxygen atoms at the terminal Si-O bonds were substituted with -CH_3_ groups to ignore the structure of the adsorption material and concentrate solely on the interplay between active groups and V_10_ species. The anchoring of the TOA fragment onto the silica surface resulted in steric hindrance. In addition, the intricate surface morphology of silica and the abundance of hydroxyl groups on the silica surface synergistically prevent the octyl chains from contacting with V_10_ species (with an enormous size), which is characterized by a substantially complicated cage configuration. DMOA, characterized by a sole octyl chain, has substantially less steric hindrance. Consequently, the V_10_ species would be more easily close to the N^+^ ionic in DMOA on the silica surface, leading to a superior adsorption efficacy. The steric hindrance significantly affects the distance between the quaternary ammonium salt and V ion, which plays a decisive role in the interaction (such as electrostatic, covalent, and dispersion). In Equation (17), ω_e_(*r*) represents the electrostatic interaction free energy between two charges, *Q*_1_ and *Q*_2_, with *r* denoting the center-to-center distance between two interacting fragments [62]. The London expression provides the formula for dispersion interactions (ω_d_(*r*), Equation (18)), where the dispersion interaction free energy varies inversely with the sixth power of the distance [62]. Covalent interactions (corresponding to orbital interactions) are short-range forces, with a range typically between 0.1 and 0.2 nm, and are significantly influenced by the distance between the quaternary ammonium salt and V_10_. Despite the TOA structure, Aliquat336 moved towards V_10_ along the path with minimal steric hindrance at the interface of the two phases in the extraction process, through intramolecular σ-bond rotation to alter the spatial configuration.

**Figure 13:**
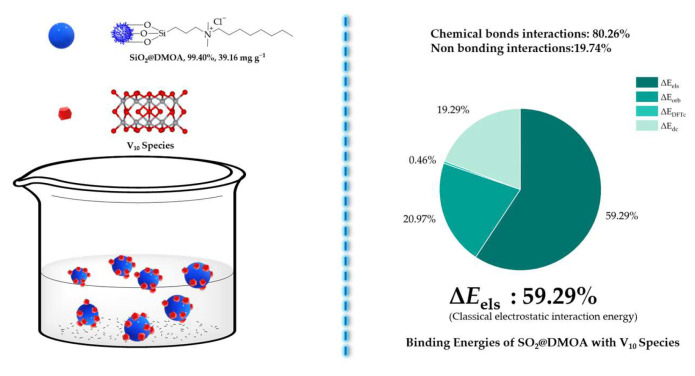
Adsorption and binding energies of SiO_2_@DMOA with V_10_ species.

The Δ*E*_int_ of DMOA-V_10_ was significantly higher than that of DMOA-HCrO_4_^−^, possibly attributable to more charges and the higher polarizability of the V_10_. By calculation, the contribution of various chemical and non-bonding interactions to Δ*E*_int_ in the sobEDA model in ranked as follows: Δ*E*_els_ (59.29%) > ΔE_orb_ (20.97%) > ΔE_dc_ (19.29%) > ΔE_DFTc_ (0.46%), as shown in Figure 13. The four types of interaction with negative values facilitate binding of the DMOA with V_10_.

(17) $$\omega_{e}\left(r\right)=\int_{\infty }^{r}-F\left(r\right)dr=-\int_{\infty }^{r}\frac{Q_{1}Q_{2}}{4\pi \varepsilon_{O}\varepsilon r^{2}}dr=+[\frac{Q_{1}Q_{2}}{4\pi \varepsilon_{O}\varepsilon r}{]}_{\infty }^{r}=\frac{Q_{1}Q_{2}}{4\pi \varepsilon_{O}\varepsilon r}$$

(18) $$\omega_{d}\left(r\right)=-\frac{3}{2}\frac{\alpha_{o1}\alpha_{o2}}{(4\pi \varepsilon_{o}{)}^{2}r^{6}}\frac{hV_{1}V_{2}}{\left(V_{1}+V_{2}\right)}=-\frac{3}{2}\frac{\alpha_{o1}\alpha_{o2}}{(4\pi \varepsilon_{o}{)}^{2}r^{6}}\frac{h{Ι}_{1}{Ι}_{2}}{\left({Ι}_{1}+{Ι}_{2}\right)}$$

It is noteworthy that the SiO_2_@TBE and SiO_2_@NTE with low steric hindrance showed a lower adsorption capacity. In the computational model, the solvent’s impact on the QAS and V_10_ was ignored. In the adsorption process, the solvation effect caused by the -OH group in the SiO_2_@TBE and SiO_2_@NTE substantially diminished the interaction between TBE/NTE substrates and V_10_. In addition, during the preparation of quaternary ammonium-functionalized silica gel, the nitrogen atoms in TBE and NTE with a weak nucleophilicity resulted in fewer functional sites in the adsorption material (compared to TMOA and TMA). From a quantum mechanical perspective, we have decomposed previously indistinct interaction energies into distinct components with clear chemical significance: electrostatic energy, Pauli repulsion, and orbital contributions. This decomposition offers initial insights into the complex relationships between bonding and non-bonding interactions characterizing the adsorption process.

## 3. Experimental Procedure

### 3.1. Reagents

All chemicals used were of analytical grade without further purification. Silica dioxide (SiO_2_, 100–200 mesh), 3-chloropropyltriethoxysilane (C_9_H_21_O_3_SiCl, 98%), trioctylamine (C_14_H_51_N, 96%), trimethylamine hydrochloride (HCl·C_3_H_9_N, 98%), *p*-tolyldiethanolamine (C_11_H_17_NO_2_, 95%), triethanolamine (C_6_H_15_NO_2_, 98%), *N*,*N*-dimethyloctylamine (C_10_H_23_N, 98%), sodium metavanadate (NaVO_3_, 96%), sodium hydroxide (NaOH, 97%), sodium chloride (NaCl, 99.5%) and acetonitrile (CH_3_CN, 99%) were purchased from Tianjin Heowns OPDE Technologies Ltd., Tianjin, China. Sodium chromate tetrahydrate (Na_2_CrO_4_·4H_2_O, 99%), toluene (C_7_H_8_, 98%), hydrochloric acid (HCl, 98%), nitric acid (HNO_3_, 98%), and sulfuric acid (H_2_SO_4_, 98%) were bought from Sinopharm Chemical Reagent Co., Ltd., Shanghai, China.

### 3.2. Synthesis of the QAS-SiO_2_

The mixture of silica dioxide (SiO_2_, 50.0 g), hydrochloric acid (375.0 mL, 12 mol/L), and distilled water (375.0 mL) was refluxed for 6 h. After cooling to room temperature, the SiO_2_ was washed with distilled water and adjusted to pH = 7, followed by drying at 130 °C for 8 h to obtain the SiO_2_@HCl.

The mixture of SiO_2_@HCl (10.0 g), 3-chloropropyltriethoxysilane (12.0 mL), and anhydrous toluene (50.0 mL) was refluxed for 24 h and centrifuged, followed by washing 5 times with a solution of ethanol and ethyl acetate (1:1). Finally, the materials were dried at 65 °C for 6 h to obtain 3-chloropropyl-functionalized silica (SiO_2_@CPTES) [63].

SiO_2_@CPTES (3.0 g) was mixed with *N*,*N*-dimethyloctylamine (5.2 g), trioctylamine (6.6 g), *p*-tolyldiethanolamine (16.5 g), triethanolamine (2.5 g), and trimethylamine hydrochloride (4.7 g), separately, in 10.0 mL of the acetonitrile. The mixture was refluxed for 8 h. The product was washed with a mixture of ethanol and ethyl acetate (1:1) to remove unreacted substances, and dried at 65 °C for 6 h to obtain a series of QAS-SiO_2_ products, such as SiO_2_@DMOA, SiO_2_@TOA, SiO_2_@TBE, SiO_2_@NTE, and SiO_2_@TMA. The synthesis route of QAS-SiO_2_ is shown in Scheme S1 in the Supplementary File.

### 3.3. Preparation of Adsorption Solution

After adjusting the pH with NaOH and HCl, various concentration of NaVO_3_, NaCl, and Na_2_CrO_4_·4H_2_O were used to prepare the adsorption solutions. The HCl, HNO_3_, and H_2_SO_4_ solutions were prepared with a concentration of 1 mol·L^−1^ for desorption.

### 3.4. Adsorption Experiment

A specific amount of the adsorbent (QAS-SiO_2_) was placed in a 50 mL centrifuge tube along with a known initial concentration of the V solution or a mixed metal solution. The mixture was stirred at 1000 rpm for 1 min to 12 h. Adsorption solution concentrations ranged from 1.0 to 8.0 × 10^−3^ mol·L^−1^, pH values ranged from 1.0 to 6.0, and the temperature ranged from 15 to 45 °C. The adsorbent mass ranged from 15 to 100 mg. After the adsorption, the mixture was filtered.

The adsorption efficiency (*A*), capacity (*q*_e_), and metal separation factor (*β*_V/Cr_) of QAS-SiO_2_ were calculated, respectively, according to Equations (19)–(22) [64]:

(19) $$A=\frac{C_{o}-C_{e}}{C_{o}}\times 100\%$$

(20) $$q_{e}=\frac{\left(C_{o}-C_{e}\right)V}{W}$$

(21) $$D=\frac{\left(C_{o}-C_{e}\right)V}{C_{e}W}=\frac{q_{e}}{C_{e}}$$

(22) $$\beta_{V/Cr}=\frac{D_{V}}{D_{Cr}}$$

where *C*_o_ (mg L^−1^) and *C*_e_ (mg L^−1^) are the initial and equilibrium concentrations of the V(V) and Cr(VI), respectively. *V* is the volume (L) of the solution, and *W* is the mass (g) of the adsorbent. *D* is the distribution ratio (L·g^−1^), and *D*_V_ and *D*_Cr_ represent the distribution ratios of V(V) and Cr(VI), respectively.

### 3.5. Desorption and Regeneration Experiment

To assess the reusability of the QAS-SiO_2_ after the V(V) adsorption, three consecutive adsorption–desorption cycles were conducted under optimal conditions. The adsorbent with loading of the V(V) was dried, mixed with different desorption agents (20 mL), stirred for 2 h at room temperature, and centrifuged. The mixture was filtered after washing to neutral pH with distilled water, followed by drying at 65 °C overnight before being used for adsorption again.

The V(V) desorption efficiency (*D*sp) was calculated according to Equation (23):

(23) $$Dsp=\left(\frac{C_{d}}{C_{o}-C_{e}}\right)\times 100\%$$

where *C*_d_ (mg·L^−1^) is the equilibrium concentration of desorbed V, and *C*_o_ (mg·L^−1^) and *C*_e_ (mg·L^−1^) are the initial and equilibrium concentrations of the V(V), respectively.

### 3.6. Characterization Techniques

Fourier transform infrared spectroscopy (FTIR, Perkin-Elmer Frontier Mid-IR FTIR/STA6000-TL9000-Clarus SQ8, Waltham, MA, USA) in a wavenumber range of 4000–400 cm^−1^ was primarily used to analyze the functional groups on the surface of silica. Before the test, the sample was fully mixed with KBr, ground, and pressed to form a thin circular disk. Scanning electron microscopy (SEM, FEI, Quanta FEG 250, Hillsboro, OR, USA) was primarily used to analyze the morphological changes in silica before and after its modification and vanadium adsorption. During this process, energy-dispersive X-ray spectroscopy (EDS) can be employed to determine the types of elements and their respective concentrations within the silica matrix. Time-of-flight mass spectrometry (TOFMS, Waters G2-IT-TOF, Milford, MA, USA) was utilized to analyze the mass of vanadium in solutions at different pH levels. Inductively coupled plasma mass spectrometry (ICP-MS, Thermo Fisher Scientific, ICAP RQ, Waltham, MA, USA) was utilized to analyze the vanadium content in vanadium solutions before and after adsorption.

## 4. Conclusions

In this study, five QAS-SiO_2_ structures were synthesized. The optimal adsorbent, SiO_2_@DMOA, was chosen for V(V) adsorption from aqueous solutions. Under optimal adsorption conditions, the V loading capacity was 39.16 mg·g^−1^. The presence of co-existing ions demonstrated that SiO_2_@DMOA remained effective in binding V(V) even at 0.5 M NaCl. Selective separation from Cr (VI) revealed that the separation factor (β_V/Cr_) increased to 135.42 at pH = 3.3. EDA analysis of a simplified interaction model showed that quaternary ammonium salts with longer carbon chains or additional hydroxyl groups can enhance binding energy with vanadium. However, in practice, steric hindrance limits the approach of bulky V_10_ species to quaternary ammonium salts with long, multiply substituted carbon chains, reducing adsorption efficiency. This model did not consider solvation effects, which contributed to the suboptimal performance of hydroxyl-containing quaternary ammonium salts in adsorption experiments. EDA also revealed that chemical bonds constitute 80.26% of the interaction energy, while non-bonding interactions make up the remaining 19.74%. Additionally, the density of quaternary ammonium salt functional groups on the silica gel surface significantly influenced the adsorption process. These findings provide a strong rationale for designing and advancing effective, highly selective adsorbents for vanadium adsorption.

## Figures and Tables

**Table 1:** Adsorption kinetic parameters for the adsorption of V(V) in various models on SiO_2_@DMOA.

Kinetic Model	Parameters	Values
Pseudo first order	*q*_e_ (mg g^−1^)	37.63
	*K*_1_ (min^−1^)	0.847
	R^2^	0.709
	RMSE	2.701
Pseudo second order	*q*_e_ (mg g^−1^)	40.33
	*K*_2_ (mg g^−1^ min^−1^)	0.0369
	R^2^	0.937
	RMSE	1.259
Elovich	*α* (mg g^−1^ min^−1^)	2599.132
	*β* (g mg^−1^)	0.237
	R^2^	0.887
	RMSE	1.686
Intraparticle diffusion	*K*_i,1_ (mg g^−1^ min^1/2^)	0.943
	*K*_i,2_ (mg g^−1^ min^1/2^)	0.490
	*C*_1_ (mg g^−1^)	19.91
	*C*_2_ (mg g^−1^)	38.08

**Table 2:** Isotherm model fitting parameters for the adsorption of V(V) onto SiO_2_@DMOA.

Langmuir					Freundlich		
*q*_m_ (mg g^−1^)	*K*_L_ (L mg^−1^)	R^2^	RMSE	*K*_F_ (L g^−1^)	1/n	R^2^	RMSE
71.30	0.0927	0.982	2.313	20.470	0.238	0.764	8.278

**Table 3:** Thermodynamic parameters.

Temperature (K)	Parameters			
	ln*K*_C_	ΔG° (kJ·mol^−1^)	ΔH° (kJ·mol^−1^)	ΔS° (J mol^−1^ K^−1^)
288	3.18	−9.81	27.08	128.08
298	3.60	−11.09		
303	3.75	−11.73		
308	3.80	−12.37		
318	4.31	−13.65		

**Table 4:** Typical parameters calculated by sobEDA analysis (kcal·mol^−1^).

	q_e_ (mg·g^−1^)	Δ*E*_int_	Δ*E*_els_	Δ*E*_x_	Δ*E*_rep_	Δ*E*_orb_	Δ*E*_DFTc_	Δ*E*_dc_
DMOA-V_10_	39.16	−27.49	−63.56	25.77	53.96	−22.48	−0.49	−20.68
TMA-V_10_	5.58	−15.69	−40.10	10.70	35.47	−12.52	−0.05	−9.19
NTE-V_10_	10.11	−32.77	−67.31	11.93	67.49	−29.00	0.09	−15.97
TOA-V_10_	2.40	−32.89	−76.63	35.38	70.50	−31.16	−0.60	−30.39
TBE-V_10_	1.19	−33.98	−68.93	12.48	70.83	−30.27	0.11	−18.20
DMOA-HCrO_4_	8.70	−12.77	−30.42	11.77	28.08	−15.51	−0.12	−6.57

## Data Availability

Data are contained within the article and Supplementary Materials.

## Supplementary Materials

The following supporting information can be downloaded at: https://www.mdpi.com/article/10.3390/molecules30071593/s1, Scheme S1. Synthesis route of the QAS-SiO_2_. Table S1. Percentage of major atoms determined by EDS. Figure S1. Time-of-flight mass spectra of V(V) (2 × 10^−3^ M) in solution at pH = 3. Figure S2. Time-of-flight mass spectra of V(V) (2 × 10^−3^ M) in solution pH 1. Figure S3. Time-of-flight mass spectra of V(V) (2 × 10^−3^ M) in solution pH 2. Figure S4. Time-of-flight mass spectra of V(V) (2 × 10^−3^ M) in solution pH 4. Figure S5. Time-of-flight mass spectra of V(V) (2 × 10^−3^ M) in solution pH 5. Figure S6. Time-of-flight mass spectra of V(V) (2 × 10^−3^ M) in solution pH 6. Figure S7. Dimensionless separation factor (RL) associated with Langmuir model.
