# Supplementary material for: New Insights into the Adsorption Mechanism of Vanadium Through Quaternary Ammonium Salt-Functionalized SiO2: Synergistic Experiments Utilizing Energy Decomposition Analysis
Source: Molecules. 2025 Apr 2;30(7):1593. doi: 10.3390/molecules30071593 (PMC11990143; doi:10.3390/molecules30071593)
Supplement: Supplementary file 1 [file molecules-30-01593-s001.zip › molecules-3501637-supplementary.pdf]

New Insights into the Adsorption Mechanism of Vanadium through Quaternary Ammonium Salt Functionalized SiO_2: Synergistic Experiments Utilizing the Energy Decomposition Analysis

Qiang Fu[1], Jianhua Tian [2], Jinjun Yang [1], Jie Wang [1], Meitong Li [1], Gangzhen Jiao [1], Yuhong Xie [1], Wenjiao Yuan [1,*], Cuihong Wang [3,*]

[1] School of Environmental Science and Safety Engineering, Tianjin University of Technology, No. 391, Binshui Xi Road, Xiqing District, Tianjin 300384, China

[2] Guangxi CNGR New Energy Science & Technology Co., LTD, Qinzhou Port Area of China (Guangxi) Pilot Free Trade Zone, Qinzhou 535035, China

[3] School of Science, Tianjin Chengjian University, No. 26, Jinjing Road, Xiqing District, Tianjin 300384, China

***Corresponding authors:**

Wenjiao Yuan, Email: wjyuan@email.tjut.edu.cn; Tel: +86-022-60214184

Cuihong Wang, Email: cuihong1231@163.com; Tel: +86-022-23085304

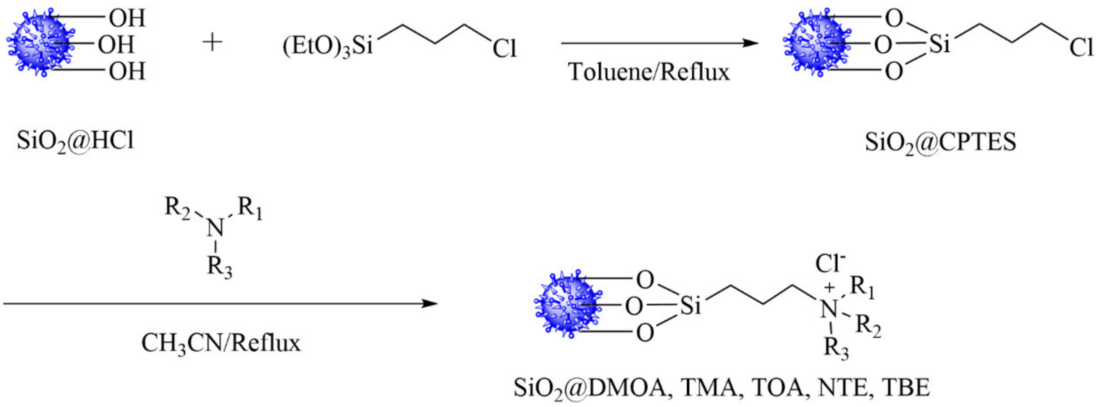

SiO_2@DMOA: R_1 = $-CH_3$, R_2 = $-CH_3$, R_3= $-C_8H_{17}$;
SiO_2@TMA: R_1 = $-CH_3$, R_2 = $-CH_3$, R_3= $-CH_3$;
SiO_2@TOA: R_1 = $-C_8H_{17}$, R_2 = $-C_8H_{17}$, R_3= $-C_8H_{17}$;
SiO_2@NTE: R_1 = $-(CH_2)_2OH$, R_2 = $-(CH_2)_2OH$, R_3= $-(CH_2)_2OH$;
SiO_2@TBE: R_1 = $-CH_2OH$, R_2 = $-CH_2OH$, R_3= -Bn.

Scheme S1. Synthesis route of the QAS-SiO_2.

Characterization

Table S1 Percentage of major atoms determined by EDS.

Sample	Detected element					
	Si	O	C	N	Cl	V
SiO_2@HCl	48.25%	51.75%	-	-	-	-
SiO_2@CPTES	21.89%	40.82%	35.12%	-	2.16%	-
SiO_2@DMOA	20.41%	39.94%	33.16%	4.96%	1.52%	-
SiO2@DMOA-Load V	21.02%	41.30%	32.36%	3.87%	-	1.45%

"-" represents no element could be detected.

S.1 Time-of-Flight Mass Spectrometry (TOF-MS)

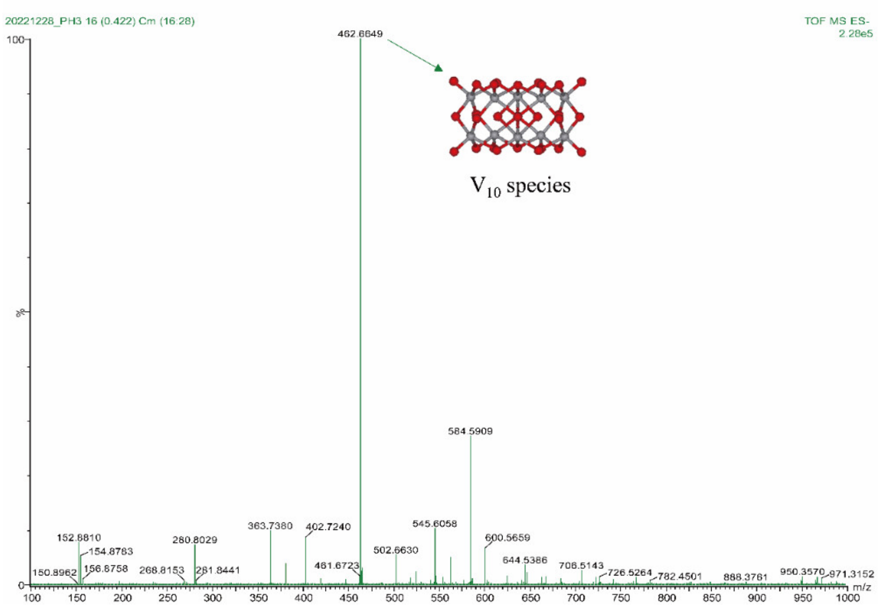

Figure. S1 Time-of-flight mass spectra of V(V) (2×10^{-3} M) in solution at pH = 3.

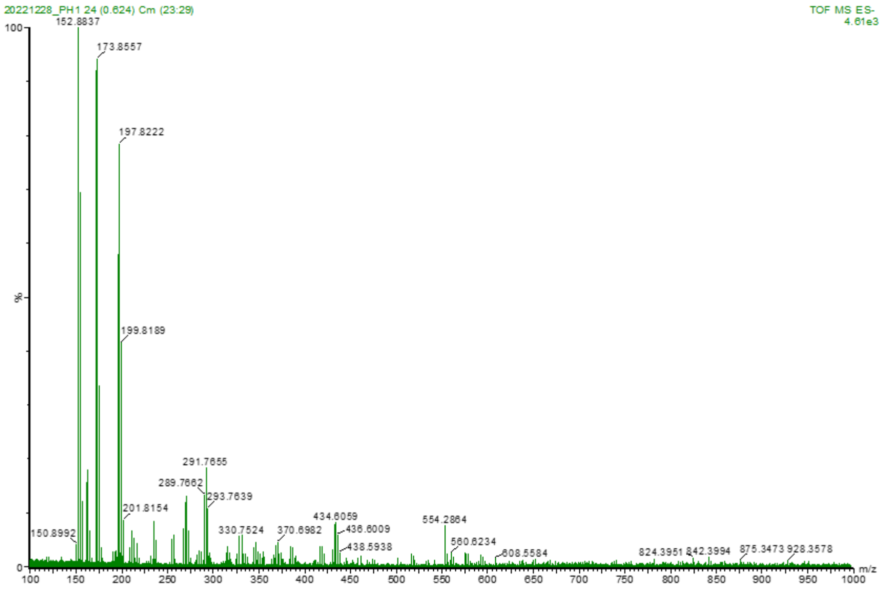

Figure S2 Time-of-flight mass spectra of V(V) (2×10^{-3} M) in solution pH 1

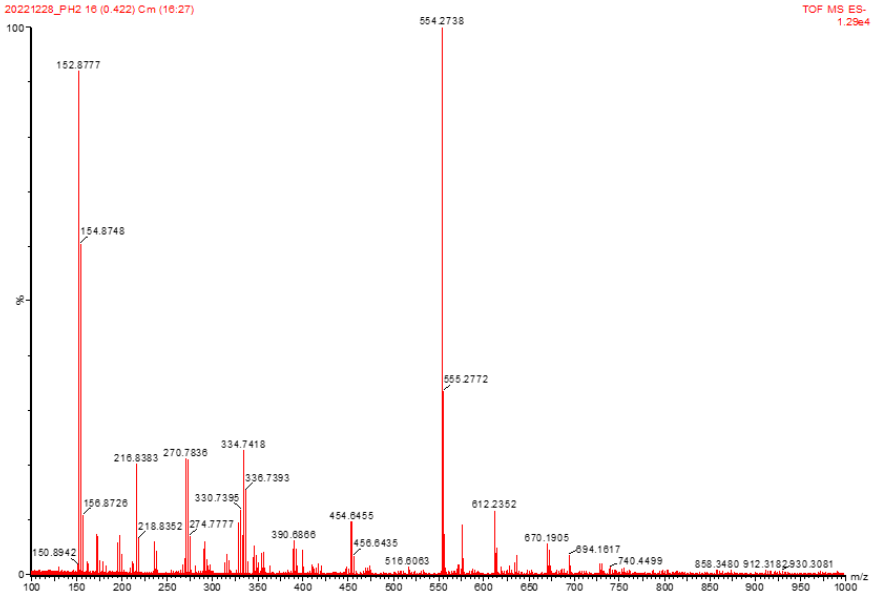

Figure S3 Time-of-flight mass spectra of V(V) (2×10^{-3} M) in solution pH 2

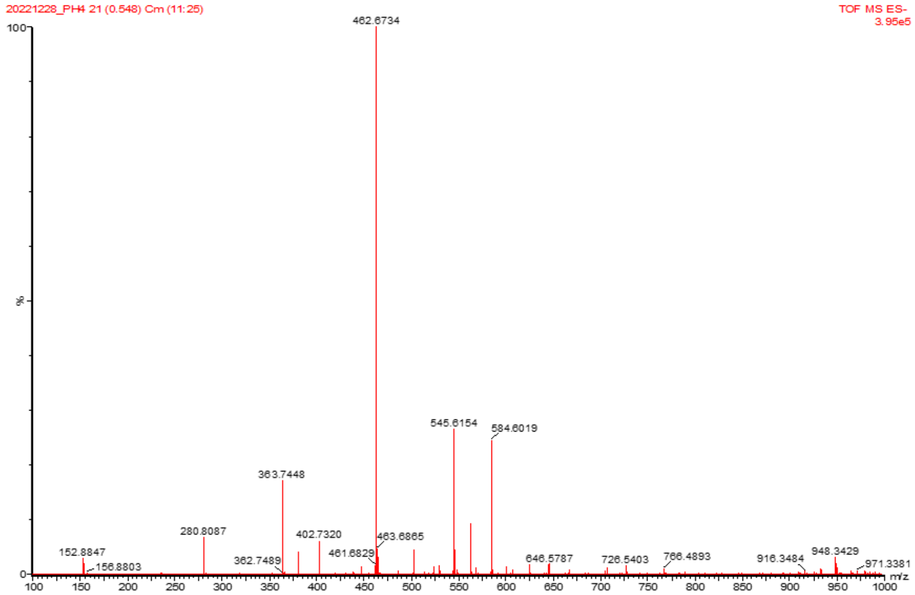

Figure S4 Time-of-flight mass spectra of V(V) (2×10^{-3} M) in solution pH 4

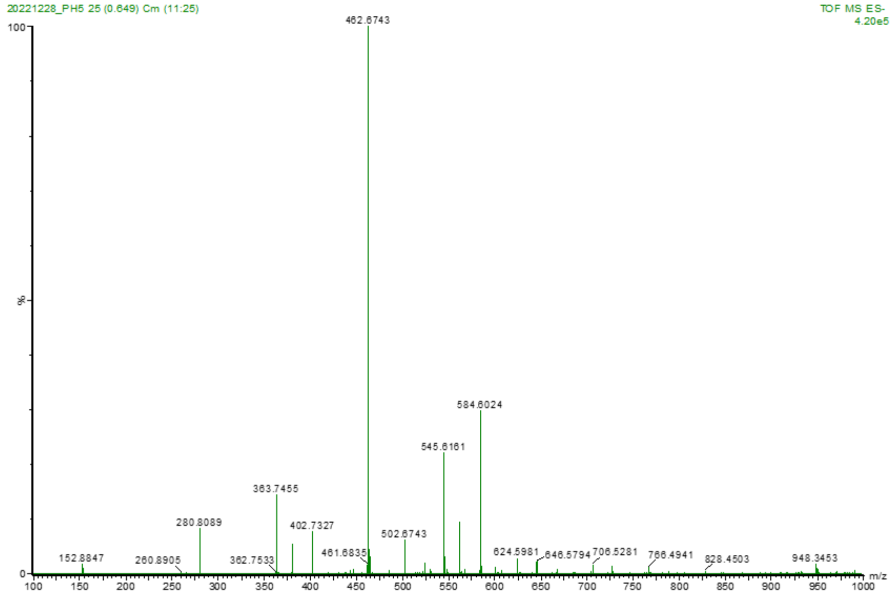

Figure S5 Time-of-flight mass spectra of V(V) (2×10^{-3} M) in solution pH 5

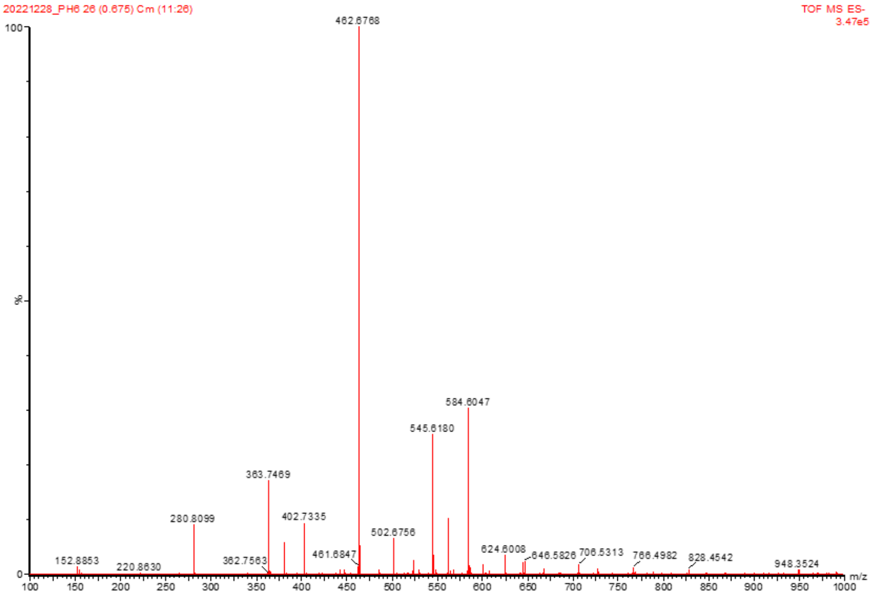

Figure S6 Time-of-flight mass spectra of V(V) (2×10^{-3} M) in solution pH 6

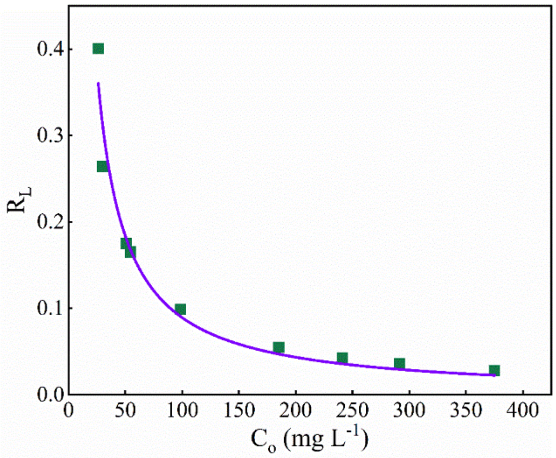

Figure. S7 Dimensionless separation factor (R_L) associated with Langmuir model.

S.2 Calculation of the total interaction energy ΔE_{int} via the sobEDA model

The computational software Gaussian16 was employed utilizing the B97D3 method. The vanadium element was described using the LanL2MB basis set, while the remaining elements were treated with the 6-31G* basis set.
